# Pleckstrin homology-like domain family A, member 3, a miR-19a-3p-regulated gene, suppresses tumor growth in osteosarcoma by downregulating the Akt pathway

**DOI:** 10.1080/21655979.2022.2031404

**Published:** 2022-02-03

**Authors:** Peng Wang, Yu Huang, Xin Xia, Jian Han, Lu Zhang, Wenzhi Zhao

**Affiliations:** a Department of Orthopedics, The Second Affiliated Hospital, Dalian Medical University, Dalian, China; b Department of Orthopedic Surgery, The Third People’s Hospital of Dalian, Non-directly Affiliated Hospital of Dalian Medical University, Dalian, China

**Keywords:** *PHLDA3*, miR-19a-3p, Akt/GSK3β, osteosarcoma, bioinformatics analysis

## Abstract

Pleckstrin homology-like domain family A, member 3 (*PHLDA3*), is emerging as a critical regulator for multiple cancers. Nevertheless, the expression and role of *PHLDA3* in osteosarcoma remain unknown. Herein, we purposed to elucidate the role of *PHLDA3* in the progression and chemoresistance of osteosarcoma. According to the bioinformatics analysis, *PHLDA3* expression was low in osteosarcoma patients, and low content was linked to poor prognosis. Additionally, activation of *PHLDA3* suppressed osteosarcoma cell proliferation, migration, and chemoresistance, whereas *PHLDA3* inhibition caused the opposite effects. Mechanistically, our data revealed that *PHLDA3* negatively regulates the Akt/GSK3β signaling cascade in osteosarcoma. Furthermore, we found that miR-19a-3p might exert its oncogenic function by inhibiting *PHLDA3* expression in osteosarcoma. These results demonstrated miR-19a-3p/ *PHLDA3*/ Akt/GSK3β axis has a pivotal role in osteosarcoma, and *PHLDA3* is a prospective therapeutic target for treating osteosarcoma.

## Graphical abstract

**Figure:**
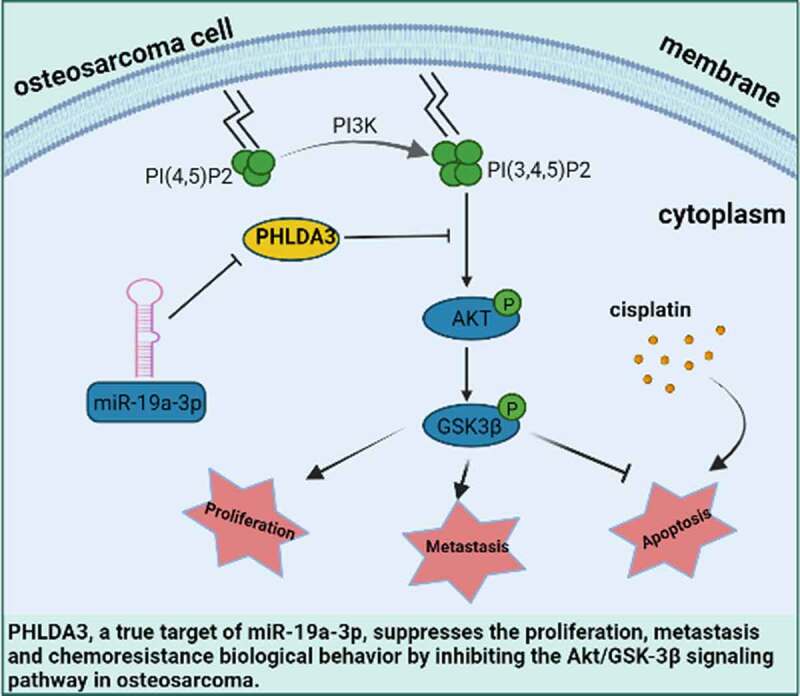

## 1. Introduction

Osteosarcoma (OS), a common bone cancer derived from mesenchymal cells, has a high incidence and mortality rate in adolescents [1,2]. It is characterized mainly by uncontrolled proliferation and a high metastasis rate: 75% of OS cases attack adjacent tissues [3]. Although several therapeutic strategies, such as surgery, adjuvant radiotherapy, and chemotherapy, have made significant advances in increasing the survival rate of OS patients, the 5-year rate of survival remains low [4,5]. Thus, it is urgent to elucidate the molecular mechanisms of OS to identify novel treatment targets.

In recent years, gene expression profiling chip, a high-throughput and effective technology, has been widely used in various fields of cancer research [6–9]. It can help in the discovery of disease-related genes and provide insight into the pathogenesis of cancers such as OS [10]. Ajimu Keremu et al. analyzed the GEO database and discovered NRSN2, a novel oncoprotein that promotes OS cell expansion and may be a prognostic biomarker of OS [11]. Hence, the gene expression profile will be helpful for us to identify reliable prognostic biomarkers to improve the prognosis and treatment of OS.

*PHLDA3* is the p53 regulatory repressor of Akt [12]. It has a PH domain that can compete with the PH domain of Akt for membrane lipids docking and suppress Akt activity [13]. Recent investigations have linked *PHLDA3* as a tumor repressor gene to diverse cancers, for instance prostate cancer [14], pancreatic neuroendocrine tumors [15], as well as breast cancer [16]. Nevertheless, the role of *PHLDA3* in OS is still unclear.

Here, we try to investigate the potential role and modulatory mechanism of *PHLDA3* in OS. Based on bioinformatics analysis, we hypothesized that *PHLDA3* is a miR-19a-3p-regulated gene and lowly expressed in human OS tissues. Additionally, we investigate the effect of *PHLDA3* on the Akt/GSK-3by blocking pathway. In summary, we aim to explore the function and its underlying mechanism of *PHLDA3* in OS and provide a new treatment strategy for OS.

## 2. Materials and methods

### 2.1 Cell culture and reagents

The human OS cell lines (143B/U2OS) were acquired from the American Type Culture Collection. The cells were cultured in Dulbecco’s Modified Eagle Medium containing 10% FBS under 37°C and 5% CO_2_ conditions. The following antibodies were used: antibody against *PHLDA3* (Abcam, ab81464); GAPDH (Santa Cruz Biotechnology, SC-25778); Actin (Proteintech,66,009-1-lg); Akt (CST, 4691); p-Akt (CST, 4060); p-GSK-3β (CST, 5558); GSK-3β (CST, 9315); and PARP (Santa Cruz Biotechnology, SC-8007).

### 2.2 Identification of the differentially expressed genes

To identify the differentially expressed genes (DEGs) in OS, the gene expression profiles (GSE42352, GSE70414) were acquired from the GEO database. The DEGs were obtained from the gene expression profiles by utilizing the GEO2R online tool (http://www.ncbi.nlm.nih.gov/geo/geo2r/). The adj. P < 0.05 and |logFC| > 1 were set as DEGs cutoff criterion.

### 2.3 Protein–protein interaction analysis

To explore the interaction of DEGs, we used the Search Tool for the Retrieval of Interacting Genes (STRING) database to analyze the protein interaction network (PPI), and the DEGs with a reliability score >0.15 were added to the Cytoscape software for the PPI.

### 2.4 Oncomine database analysis

Oncomine database (http://www.oncomine.org), a web-based microarray database, was used to analyze the transcription level of the DEGs in different cancer types. PHLDA3 gene expression in clinical cancer tissue was queried and compared that with normal tissue using Student t-test. The parameters included fold-change 2, P-value 1e-4, and gene rank top 10%.

### 2.5 UCSC Xena

The prognostic value of PHLDA3 expression in OS was assessed according to overall survival using the UCSC Xena browser (http://xena.ucsc.edu/), an online database including gene expression data and clinical data.

### 2.6 Transfection and viral infection

The Lipofectamine™3000 system was adopted to perform transient transfections (Invitrogen) as described by the manufacturer. The miR-19a-3p mimics and inhibitors were acquired from GenePharma. The short hairpin RNA (shRNA) sequences targeting *PHLDA3* were 5’-AGCGCTGCGTCCTCACCGA-3’ and 5’- GCGACGGCTACCGTGCTCA −3’. The shRNA was cloned via the pGIPZ vector. Human *PHLDA3* cDNA was cloned into the pCDH vector using the primers: F: 5’- GCGAATTCATGACGGCGGCGGCGACGG-3’ and R: 5’- GCGGATCCTTAGGACACGAGGGTCCCGGTC-3’. The expression efficiency was evaluated using Western blot analysis.

### 2.7 MTT assay and PI staining assay

Briefly, 3000 cells were inoculated into 96-well plates. At the specified point in time, 200 μl culture media containing 20 μl MTT was introduced into each well and left to grow for four hours. Afterward, we replaced the medium in every well with 200 μl DMSO. The OD values at 570 and 630 nm were read with a microplate reader (PerkinElmer).

For propidium iodide (PI) staining assay, OS cells were inoculated in 6-well plates and exposed to or without cisplatin (CDDP) for 24 h. The cells were then stained with PI (Coolaber, cp9161) and left to grow for 10 minutes (Coolaber, cp9161). Finally, the morphologic cell changes were recorded using a fluorescence microscope (Olympus).

### 2.8 Colony formation assay

We trypsinized the 143B/U2OS cells, and then sub-cultured 1000–3000 viable cells in 6-well plates and left the cells to grow for 14 days for adherence and colonization. After that, we removed the media cells, then stained the cells (in 0.04% crystal violet) to visualize colonies and assess proliferation capacity.

### 2.9 Sphere formation assay

Suspensions of cells were inoculated on ultra-low adherent 12 well plates at 1 × 103 cells per well in 1 mL serum-free DMEM-F12 medium (Gibco, MA) enriched with B27 (1:50) (Invitrogen, MA) along with 20 ng/ml bFGF and EGF. After 14 days of incubation, the number of spheres exhibiting a diameter > 100 mm in each well was recorded.

### 2.10 Cell migration assay

In compartments (BD, Biosciences, 8.00 mm pores), 143B/U2OS cells were inoculated in serum-free medium. We left the cells to migrate toward the serum-rich bottom chamber for 24 hours. Non-migrating cells were scraped off the insert’s apical surface, and membranes harboring migrated cells were fixed (in 4% paraformaldehyde), followed by staining (in crystal violet). The cells’ average count per field of view was used to express cell count.

When the transfected 143B and U2OS cells grew to more than 90% of their original size on the 12-well plate, a linear wound was made with a sterile 200-μl pipette tip. The cell fragments were rinsed thrice with PBS, before being inoculated in 2% FBS medium. The 12-well plate was photographed under a microscope. The cell migration images were taken after 16/24/36 h at a fixed position.

### 2.11 Real-time RT-PCR

Isolation of total RNA was done with Trizol (Invitrogen). Afterward, cDNA was generated from 1 μg of total RNA with the PrimeScriptTM RT Kit as described by the manufacturer (Takara, RR047A). The following primers were used: PHLDA3: F: 5’-CAGCTCTTCGAGGCCAAG-3’ and R: 5’-GACCAGGCCTAGGGTGATCT-3’; Actin: F: 5’-GACCTGACTGACTACCTCATGAAGAT-3’ and R: 5’-GTCACACTTCATGATGGAGTTGAAGG-3’.

### 2.12 Annexin V-FITC staining and flow cytometry

Transfected 143B cells were trypsinized and collected in 300 μl of the provided Annexin V-FITC (AV) binding buffer. Cells were then co-inoculated in the dark with 5 μl AV and 10 μl PI for 10 minutes at RT (room temperature). Thereafter, we suspended the cells in binding buffer (400 μl) followed by gentle mixing. Lastly, the treated cells were subjected to flow cytometry (BD Biosciences).

### 2.13 Dual-luciferase reporter assay

The dual-luciferase enzyme reporter assay was conducted as previously documented [17]. The wild-type (WT) docking site of miR-19a-3p on PHLDA3 and the matching mutant (Mut) were cloned into the pGL3-basic vector. The PHLDA3 WT or PHLDA3 Mut was co-transfected into 143B/U2OS cells with miR-19a-3p mimics or inhibitor or their corresponding control. After 48 h, Promega Dual-Luciferases Reporter Assay kit (Promega E1980) was performed to measure luciferase activities according to the manufacturer’s protocols.

### 2.14 Animal experiments

Animal experiments were conducted according with the National Institute of Health Guide for the Care and Use of Laboratory Animals with the approval of the Animal Research Committee of Dalian Medical University. 143B cells transfected with sh-PHLDA3 or shCtrl were injected into the flank of the nude mice. The mice were killed and tumors were removed from the mice at 18 days after inoculation.

### 2.15 Statistics and data analyses

The data are given as mean ± SD. The statistical assessment was done using one-way analysis of variance (ANOVA), with *p* < 0.05 signifying statistical significance.

## 3 Results

In the study, we aimed to elucidate the molecular mechanisms of OS to identify novel treatment targets. Based on bioinformatics analysis, we discovered that *PHLDA3* expression was low in OS cells and tissues. Increased *PHLDA3* in OS cells suppressed cell growth, migration, and enhanced cisplatin-triggered apoptosis. Whereas, *PHLDA3* knockdown increased cell growth, migration, and dampened cell apoptosis. Furthermore, we established that *PHLDA3* was a true target of miR-19a-3p and blocked the Akt/GSK-3β signaling cascade in OS cells.

### 3.1 PHLDA3 *expression was downregulated in osteosarcoma cells and tissue samples*

**Figure 1.**
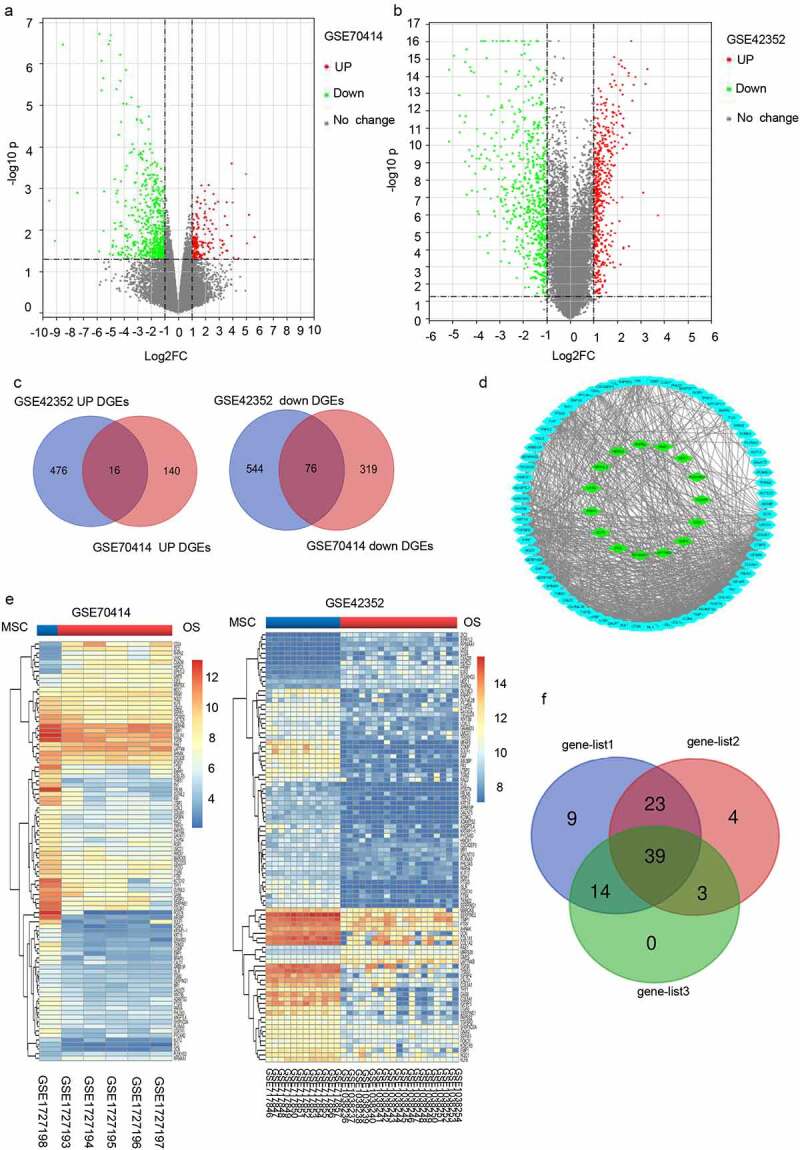
Differentially expressed mRNAs between osteosarcoma cell and mesenchymal stem cells. (a-b) The mRNA Volcano plot based on (a) GSE70414 data and (b) GSE42352 data. The red points designate upregulated genes screened as per |fold change|>1.0 and *p* < 0.05. The green points designate downregulated genes screened as per |fold change|>1.0 and *p* < 0.05. The gray points show genes with no statistically remarkable difference. (c) Venn diagrams of the overlapping DEGs between the two datasets. (d) Protein-protein interaction (PPI) networks for 92 differentially co-expressed genes. Green and blue nodes denote upregulated and downregulated genes, respectively. (e) Heat map showing 92 differentially co-expressed genes, with blue to red colors representing mRNA expression levels from low to high. (f) 23 valuable genes screened based on literature search. Blue, red, and green circles indicate genes that are rarely reported with osteosarcoma (<5), genes with more reported tumors (>10), and genes with the opposite trend of tumor reports, respectively.

To determine the differentially expressed genes (DEGs) in OS, we obtained the associated expression microarray datasets from GEO databases, including GSE70414 and GSE42352. The two datasets were evaluated using the GEO2R data resource (http://www.ncbi.nlm.nih.gov/geo/geo2r/) to determine DEGs (|Log FC|> 1 and *p*-value< 0.05) between OS cells and bone mesenchymal stem cells (MSC). Volcano plots were used to display the DEGs from the two sets of each sample data (Figure 1a and b). A total of 551 and 1112 DEGs were obtained from GSE70414 and GSE42352 datasets, respectively. Individually, the Venn diagram revealed 16 common upregulated genes and 76 common downregulated genes (Figure 1c). The heat maps of the 92 differentially co-expressed genes in various chips were generated using the ‘pheatmap’ package in R studio (Figure 1e). The differentially co-expressed genes were then imported into the STRING database, and those with a reliability score >0.15 were added to the Cytoscape software for the visual protein interaction network (Figure 1d).

**Figure 2.**
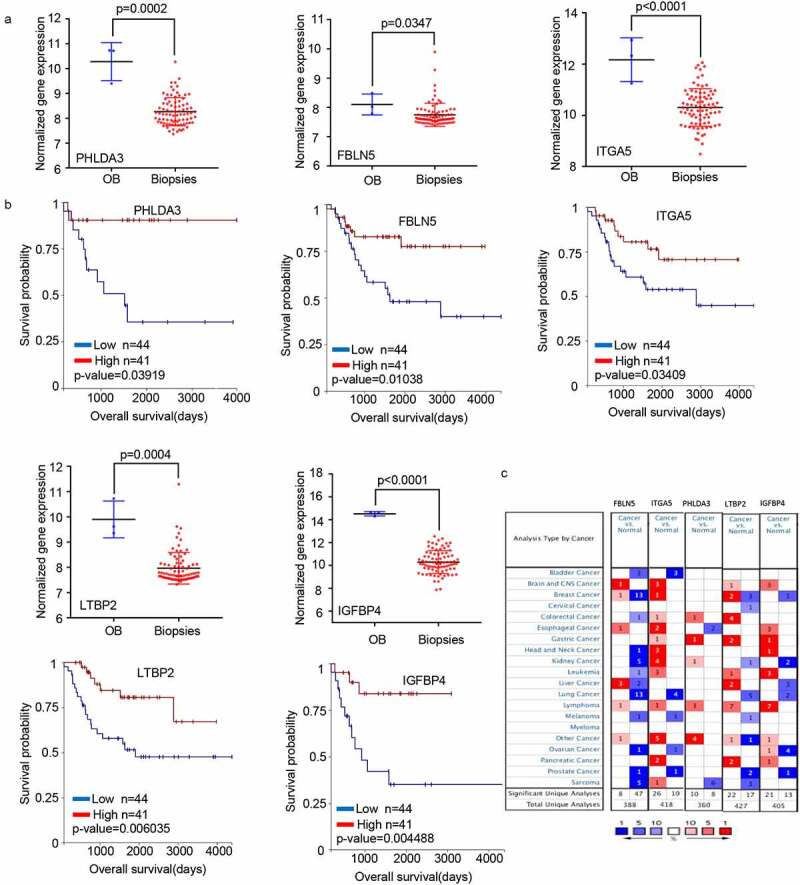
The mRNA expression levels of five genes screened from 23 valuable genes. (a) Normalized gene expression levels of the five screened genes in osteoblasts (OB) and osteosarcoma biopsies (Biopsies). (b) The prognostic value of mRNA contents of the five screened genes in osteosarcoma patients (UCSC). (c) Transcription levels of the five screened genes in different types of cancers (Oncomine).

We selected 23 genes from a literature search to screen for those with potential research value (Figure 1f). Then, we extracted the expression profiles of the selected genes from GSE42352 datasets between osteoblast (OB) and high-grade OS pre-chemotherapy biopsy (Biopsies) and analyzed the influence of these genes on the clinical survival of patients with OS using UCSC Xena browser (http://xena.ucsc.edu/). We found that the changing trend of five genes (*PHLDA3, FBLN5, ITGA5, LTBP2*, and *IGFBP4*) in Biopsies and OB was consistent with OS cells and MSC outcomes (Figure 2a), and affected the prognosis of OS patients (Figure 2b). The transcriptome analysis of the five genes in different types of tumors in the Oncomine data resource (https://www.oncomine.org/resource/login.html) revealed that the expression of *PHLDA3, FBLN5*, and *LTBP2* was low in clinical samples of sarcoma (Figure 2c). Based on additional verification data sets, we eventually selected *PHLDA3* as a candidate gene for further experimental verification.

### 3.2 PHLDA3 *inhibits osteosarcoma cell proliferation and migration*

**Figure 3.**
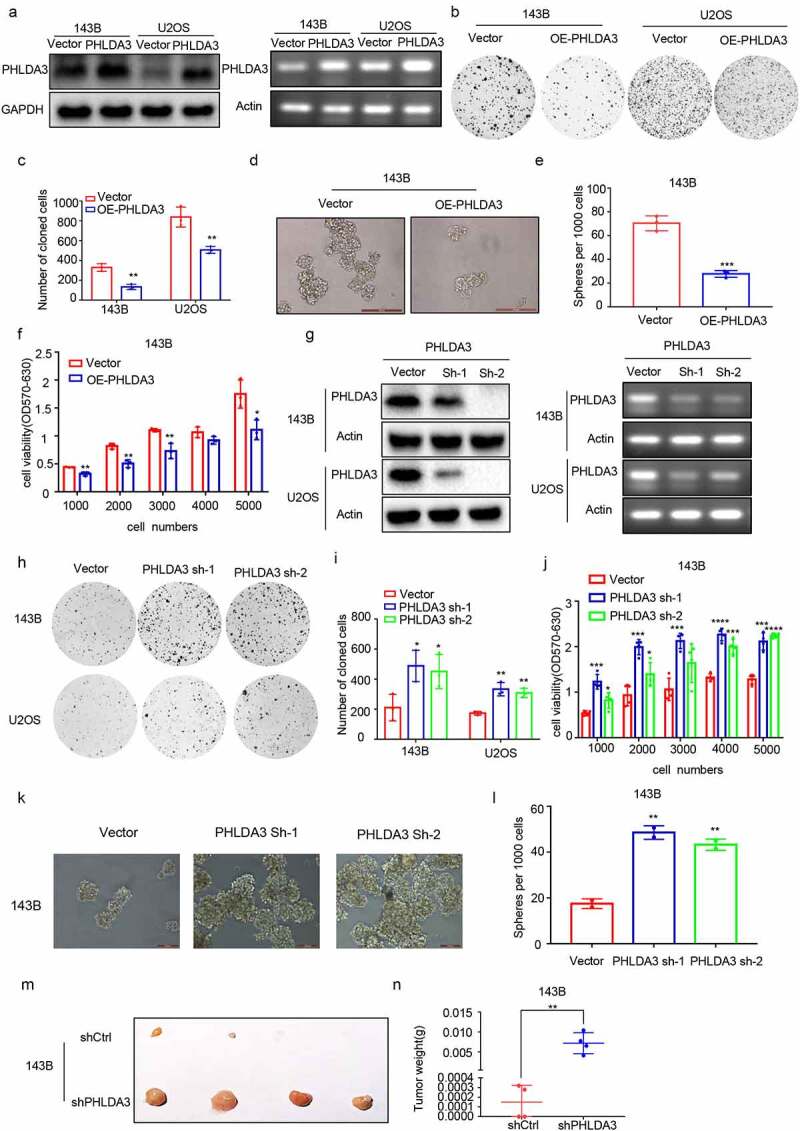
*PHLDA3* suppressed the proliferation of human osteosarcoma cell lines. (a) Relative protein and mRNA contents of *PHLDA3* in 143B/U2OS cells after transfection with OE-PHLDA3, as confirmed using Western blot and RT-PCR. (b-c) Cell growth ability of 143B/U2OS cells after transfection based on colony formation assays. (d-e) Tumor-initiating capability of 143B cells after transfection, as assessed using sphere formation assay. (f) Cell viability of 143B cells after transfection based on MTT assays. (g) Knockdown efficiency of *PHLDA3* using shRNA lentivirus. *PHLDA3* expression levels in *PHLDA3* knockdown (shPHLDA3-1 and shPHLDA3-2) and negative control cells, as detected using Western blot and RT–PCR. (h-i) Colony formation assays. (j) MTT assays. (k-l) Sphere formation assay. (m) The tumor size of nude mice. (n) Tumor weighs of Xenograft tissues. The data in (c), (e), (F), (i), (J), (l) and (N)were given as means ± SD, *p < .05, **p < .01, ***p < .001 vs. control.

The biological role of *PHLDA3* in OS is poorly understood. Therefore, we overexpressed *PHLDA3* (OE-PHLDA3) in 143B and U2OS cells to explore its role in OS. The predicted level of *PHLDA3* expression was verified using Western blot along with RT-PCR (Figure 3a). After that, we explored the influence of *PHLDA3* on cell proliferation using MTT, colony formation, and spheroid formation assays. We found that increased *PHLDA3* remarkably dampened the growth of OS cells, as illustrated in Figure 3b-f (*p* < 0.05). To verify this finding, we used lentiviruses expressing shRNA to knock down *PHLDA3* expression. The knockdown efficiency was assessed using Western blot and RT-PCR (Figure 3g). The results indicated that silencing *PHLDA3* significantly increased cell proliferation (Figure 3h-l, *p* < .05). Besides, sh-PHLDA3 or shCtrl transfected 143B cells were inoculated into nude mice to assess the functional role of *PHLDA3* in vivo. As shown in Figure 3m-n, *PHLDA3* silencing greatly increased the tumor weight of mice.

**Figure 4.**
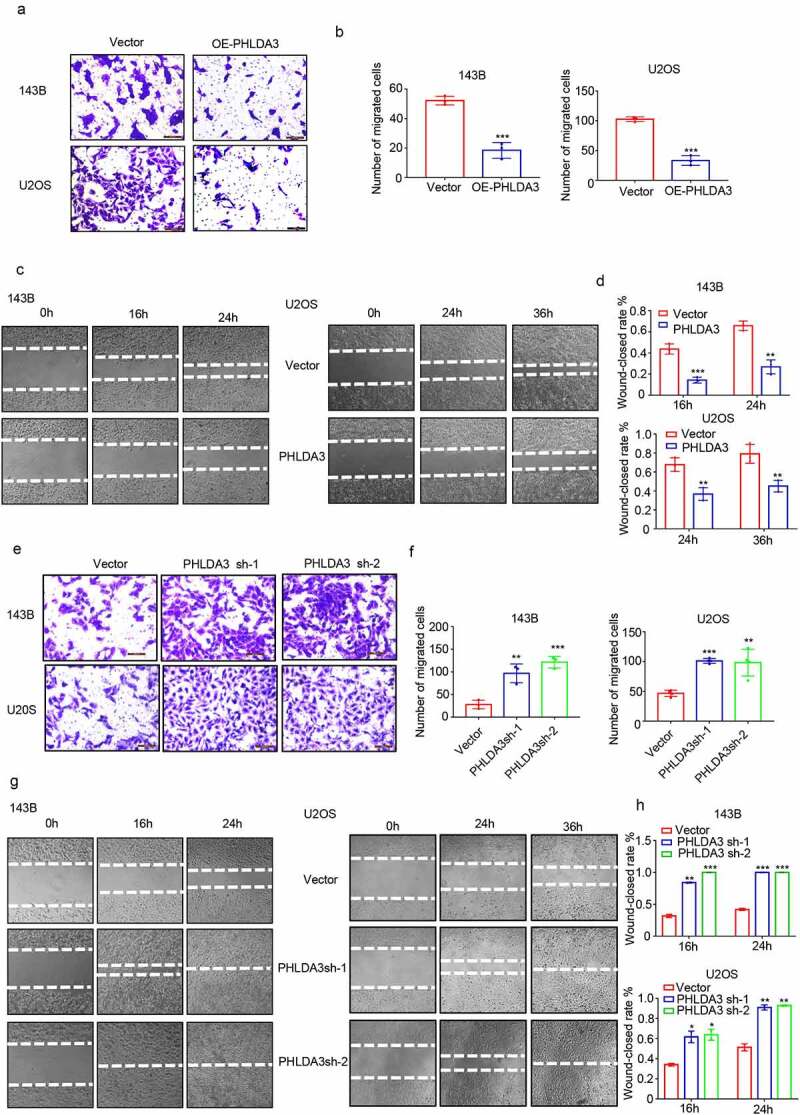
*PHLDA3* suppressed cell migration in osteosarcoma. (a-d) Overexpression of *PHLDA3* in 143B/U2OS cells via lentivirus vectors. The influences of *PHLDA3* on cell migration were explored via Transwell assay (a-b) along with wound-healing assay (c-d). (e-h) *PHLDA3* knockdown in 143B/U2OS cells, as well as its influence on cell migration were explored via Transwell assay (e-f) along with wound-healing assay (g-h). The data in (b), (d), (f), and (h) were given as means ± SD, **p < .01, ***p < .001 vs. control.

Subsequently, the effect of *PHLAD3* on OS cell migration was assessed via wound healing along with Transwell assays. The results indicated that increased *PHLDA3* suppressed OS cell migration (Figure 4a-d, *p* < .01), whereas *PHLDA3* knockdown accelerated cell migration in 143B/U2OS cells (Figure 4e-h, *p* < .01). Collectively, these results illustrate that *PHLDA3* is a *bona fide* repressor of cell proliferation along with migration in OS.

### 3.3 PHLDA3 *enhances cisplatin-induced apoptosis in osteosarcoma cells*

**Figure 5.**
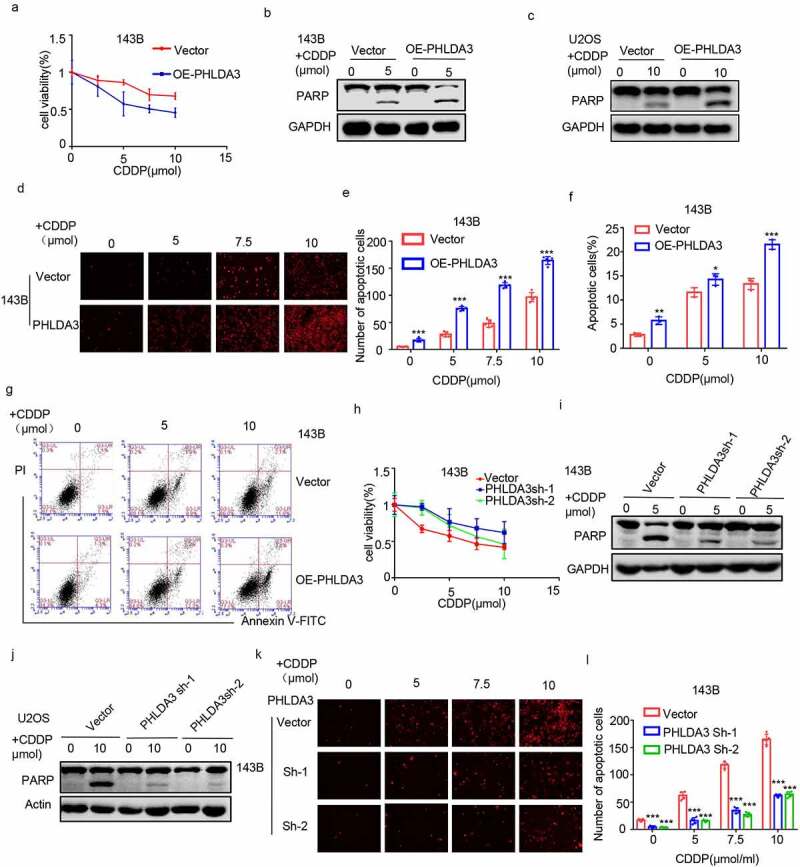
*PHLDA3* enhanced cisplatin-triggered apoptosis in osteosarcoma cells. (a) The transfected 143B cells were exposed to different doses of cisplatin (CDDP) for 24 hours, and cells viability was explored via the MTT assay. (b-g) Osteosarcoma cells were inoculated with diverse levels of CDDP for 24 hours after OE-PHLDA3 transfection. Cell apoptosis was analyzed using Western blotting analysis of PARP (b-c), PI only staining (d-e), and Annexin V/PI double staining (f-g). (h) The transfected 143B cells were exposed to diverse levels of CDDP for 24 hours, and cells viability was explored via the MTT assay. (i-l) Osteosarcoma cells were inoculated with diverse levels of CDDP for 24 hours after Sh-PHLDA3 transfection. Cell apoptosis was analyzed using Western blotting analysis of PARP (i-j) and PI only staining (k-l). The data in (e), (f), and (l) were given as means ± SD, ***p < .001 vs. control.

To confirm the tumor-suppressive role of *PHLDA3*, we assessed its effect on chemoresistance in OS cells. We found that overexpression of *PHLDA3* promoted cisplatin-induced apoptosis, as illustrated by the escalation in cleaved PARP (Figure 5b-c). Subsequently, these findings were corroborated by the MTT (Figure 5a), PI staining (Figure 5d-e, *p* < 0.001), and flow cytometry analyses (Figure 5f-g, *p* < 0.05). According to the results, overexpressing *PHLDA3* reduced cell viability, as well as elevated the numbers of cell apoptosis in OS cells treated with cisplatin. To verify the results, we silenced *PHLDA3* in OS cells, and assessed cell viability and apoptosis using MTT assay (Figure 5h), Western blot (Figure 5i-j), and PI staining (Figure 5k-l, *p* < 0.001). *PHLDA3* knockdown consistently increased OS cells viability and decreased cells apoptosis in response to cisplatin treatment. Hence, these data illustrated that *PHLDA3* plays an indispensable role in the chemoresistance in OS cells.

### 3.4 *PHLD*A3 suppresses cell proliferation and migration via Akt/GSK-3β signaling in osteosarcoma cells

**Figure 6.**
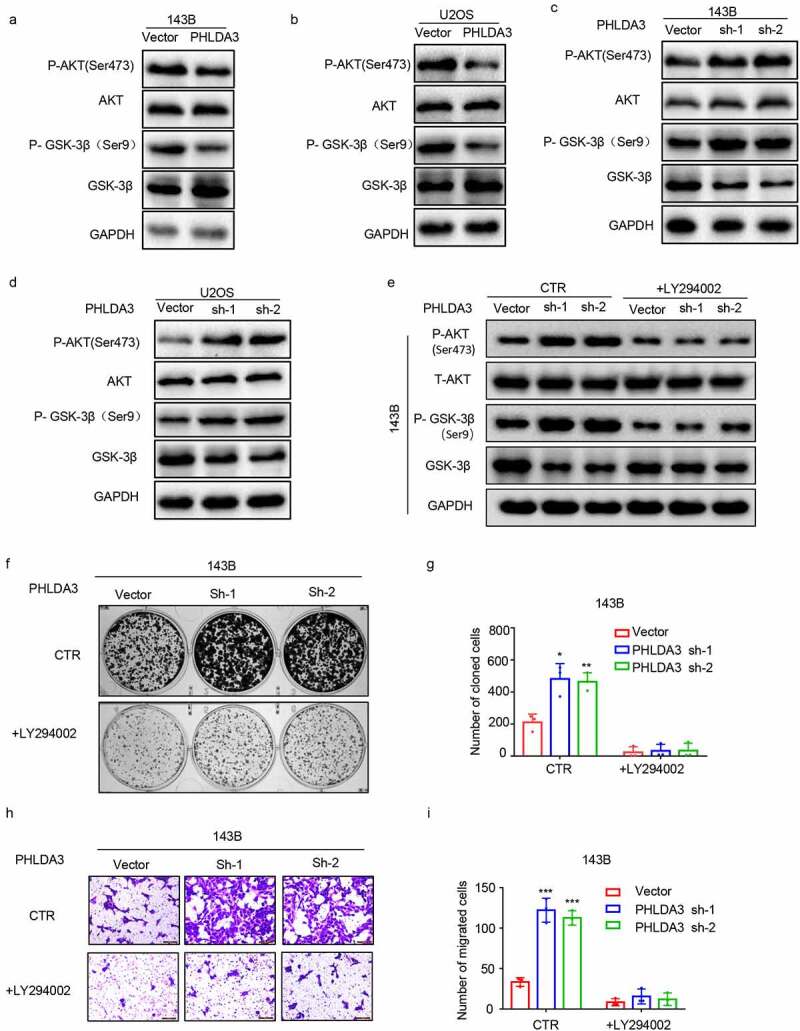
Akt/ GSK-3β signaling cascade is regulated by the expression of *PHLDA3*. (a-d) Western blots for Akt, GSK-3β, p-Akt along with p-GSK-3β protein contents in 143B/U2OS cells after transfection. (e) Protein contents of Akt, GSK-3β, p-Akt, and p-GSK-3β in 143B cells after inoculation with *PHLDA3* downregulation and/or LY294002 (20 μmol) for 24 h. (f-g) The proliferation of 143B cells after treatment with *PHLDA3* downregulation and/or LY294002 (20 μmol) examined using a colony formation assay. (h-i) The migration of 143B cells after treatment with *PHLDA3* downregulation and/or LY294002 (μmol) examined using Transwell assays. The data in (g) and (i) were given as means ± SD, ***p < .001 vs. control.

Akt signaling is well recognized for its pivotal role in enhancing cell growth and dampening apoptosis in many cancers [18]. Previous studies indicate that *PHLDA3* is an important suppressor of Akt signaling [19]. Therefore, to investigate whether *PHLDA3* regulates OS cell proliferation along with migration through the Akt/GSK-3β cascade, we first assessed its effect on p-AKT and p-GSK-3β protein contents in OS cells. The results illustrated that *PHLDA3* overexpression diminished the contents of p-AKT (ser 473) along with p-GSK-3β (ser 9) (Figure 6a-b), whereas *PHLDA3* knockdown elevated the protein contents of p-AKT (ser 473) coupled with p-GSK-3β (ser 9) (Figure 6c-d).

To demonstrate that *PHLDA3* suppressed cell growth along with migration by regulating the Akt cascade, OS cells with or without *PHLDA3* silencing were inoculated with or without the PI3K/Akt signaling inhibitor LY294002. The data indicated that LY294002 remarkably diminished the p-AKT and p-GSK-3β induced by *PHLDA3* knockdown (Figure 6e). Furthermore, cells proliferation along with migration were evaluated via colony formation coupled with Transwell assays. LY294002 prevented the increase of cell proliferation (Figure 6f-g, *p* < .05) and migration (Figure 6h-i, *p* < .001) caused by *PHLDA3* knockdown. According to the results, *PHLDA3* suppresses OS development by modulating the Akt/GSK-3β signaling cascade.

### 3.5 PHLDA3 *is a direct downstream target of miR-19a-3p*

**Figure 7.**
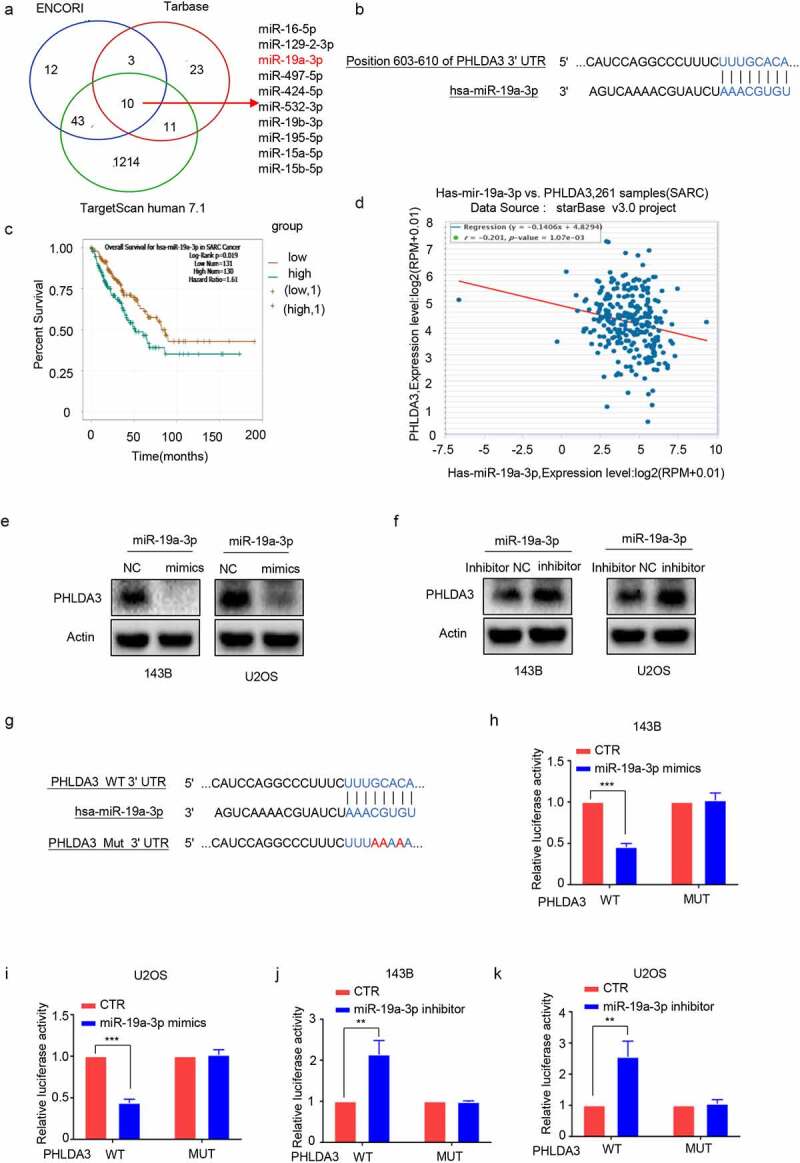
*PHLDA3* was a direct target of miR-19a-3p. (a) The candidate *PHLDA3* miRNAs as predicted using online databases (ENCORI, TargetScan, and Tarbase). (b) The binding sites of *PHLDA3* and miR-19a-3p as predicted by online TargetScan software. (c) High miR-19a-3p gene expression predicted a poorer survival outcome for sarcoma patients, as shown by the ENCORI software analysis. (d) Negative relationship of miR-19-3p content with *PHLDA3* levels computed from sarcoma samples. (e-f) MiR-19a-3p inversely modulated *PHLDA3* expression in 143B/U2OS cell lines, as explored via Western blot. (g) Schematic illustration of luciferase reporters harboring the wild-type docking site of miR-19a-3p on *PHLDA3*, named *PHLDA3* wild type (*PHLDA3* WT), and the mutant docking site, named *PHLDA3* Mut. (h-k) *PHLDA3* WT or Mut together with the miR-19a-3p mimics or inhibitor were transfected into 143B/U2OS cells. Luciferase activity was explored.

To understand the modulatory mechanism of *PHLDA3* in OS, we searched for potential *PHLDA3* miRNAs in three databases: ENCORI, TargetScanHuman7.1, and Tarbase. The miRNAs were then verified using a PubMed literature review. The data illustrated that miR‐19a-3p, a predicted miRNA of *PHLDA3*, was upregulated in OS [20,21] (Figure 7a). Thus, we purposed to establish the association of miR-19a-3p with *PHLDA3*. Subsequently, Target ScanHuman7.1 predicted that there was a docking site for miR-19a- 3p in the 3′ UTR of *PHLDA3* (Figure 7b). The relationship of miR‐19a-3p expression with the survival outcomes in sarcoma patients was analyzed using ENCORI. The data illustrated that a low miR‐19a-3p level may estimate a better survival outcome (Figure 7c, *p* < 0.05). Furthermore, there was a clear negative association of miR-19a-3p expression with *PHLDA3* expression in sarcoma cancer samples (Figure 7d, *p* < 0.01), as well as a negative modulation of miR-19a-3p on *PHLDA3* expression in 143B/U2OS cells (Figure 7e-f). The analysis of luciferase reporter genes revealed a direct cross-talk of miR-19a-3p with the 3'UTR of *PHLDA3* (Figure 7g-k, *p* < 0.01). These data illustrate that *PHLDA3* is a direct target of miR-19a-3p.

**Figure 8.**
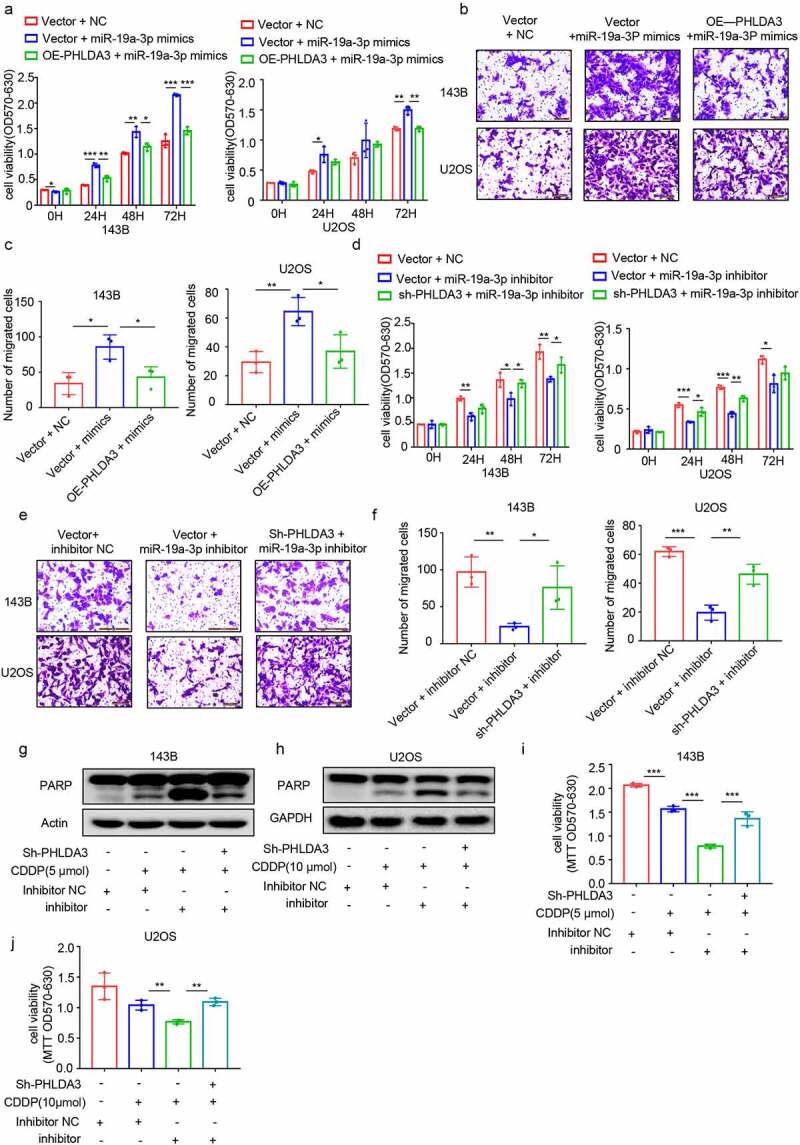
MiR-19a-3p exerted its role on cell proliferation, metastasis, and chemo-sensitivity via dampening *PHLDA3* expression. (a-c) The 143B/U2OS cells were inserted with miR-19a-3p mimic or NC via transfection for 24 h. (a) Cell viability based on MTT. (b-c) The migration of osteosarcoma cells examined using Transwell assays. (d-f) The 143B/U2OS cells were inserted with miR-19a-3p inhibitor or inhibitor NC via transfection for 24 h. (d) Cell viability based on MTT. (e-f) The migration of 143B/U2OS cells as explored via Transwell assay. (g-j) Transfected 143B/U2OS cells were untreated or inoculated with cisplatin (CDDP) for 24 h. Cell apoptosis was analyzed using Western blotting analysis of PARP (g-h) and MTT (i-j). The data in (A), (c), (D), (f), (i), and (j) were given as means ± SD, *p < .05, **p < .01, ***p < .001 vs. control.

To confirm whether the effects of miR-19a-3p on OS cell proliferation along with migration were *PHLDA3*-dependent, we transfected miR-19a-3p into OS cells with or without *PHLDA3* overexpression or knockdown. The MTT (Figure 8a, *p* < .05) and Transwell assays (Figure 8b-c, *p* < 0.05) results illustrated that restoration of *PHLDA3* prevented the miR-19a-3p-induced increase in cell proliferation and migration. In contrast, *PHLDA3* suppression prevented the miR-19a-3p inhibitor from decreasing cell proliferation (Figure 8d, *p* < .05) and migration (Figure 8e-f, *p* < 0.05).

Furthermore, to investigate whether miR-19a-3p regulated chemoresistance in OS cells via suppressing the *PHLDA3*/Akt pathway, we evaluated cell apoptosis using Western blotting (Figure 8g-h) and MTT (Figure 8i-j, *p* < 0.01). We observed that miR-19a-3p repressor increased chemosensitivity in 143B/U2OS cells, which was reversed by *PHLDA3* knockdown. These data illustrated that miR‐19a‐3p has an oncogenic effect on OS cells by directly targeting *PHLDA3*.

## 4. Discussion

*PHLDA3* is a novel tumor-related protein that mediates carcinogenesis of multiple cancers. However, the relevance of *PHLDA3* in OS is still unknown. In this study, we explored the role of *PHLDA3* in osteosarcoma and investigated the involvement of potential signaling pathway. We verified that *PHLDA3* played a tumor suppressor role in osteosarcoma and suppressed cell proliferation, migration and enhanced cisplatin-induced cell apoptosis via regulating the Akt/ GSK-3β signaling pathway. Additionally, our data indicated that *PHLDA3* was a true target of miR-19a-3b and miR-19a-3p promoted OS cell growth and migration by inhibiting *PHLDA3* expression.

*PHLDA3* was initially recognized as a p53 target gene with only a PH domain that competes with Akt. Accumulating studies have identified *PHLDA3* as a vital tumor suppressor gene that is linked to tumorigenesis, disease progress along with poor prognosis in various human cancers. For example, decreased *PHLDA3* expression was remarkably linked to tumor progress along with recurrence in individuals with squamous cell carcinomas, indicating a poor prognosis [22]. Loss of heterozygosity (LOH) at the *PHLDA3* gene locus is linked to the progress and malignant phenotype of pancreatic neuroendocrine tumors (PanNETs) [13]. Moreover, several studies have demonstrated that *PHLDA3* is differentially expressed in cisplatin-induced acute kidney injury patients [23,24]. In addition, *PHLDA3* could regulate energy metabolism [19], impede somatic cell reprogramming [25], and ameliorate pressure overload-triggered cardiac remodeling [26]. Consistently, we proved that *PHLDA3* inhibited OS cell proliferation, migration and enhanced cisplatin-triggered cell apoptosis. Hence, our findings also revealed that *PHLDA3* is a tumor repressor gene in OS and elevated *PHLDA3* may be beneficial to decrease tumor size and clinical nursing of patients with OS.

As a PH domain-only protein, *PHLDA3* may compete with Akt, another PH-domain-containing protein, for membrane lipid binding, thereby interfering with Akt translocation to the cellular membrane and activation. Most of the biological functions of *PHLDA3* have been linked to blocking Akt signaling via its PH domain. It has been documented that *PHLDA3* regulates the Akt/GSK3β signaling during somatic cell reprogramming [25]. As expected, we established that the suppressor role of *PHLDA3* in OS was still exerted by suppressing the Akt-GSK3β signaling. The Akt/GSK-3β signaling is a widely-known indispensable controller in different biological processes of the multifarious types of tumor cells [27–30]. Accumulating evidence indicates that Akt/GSK-3β is linked to osteosarcoma progression [31] and anti-chemotherapeutic drugs [32]. Therefore, the role of the PHLDA3/Akt/GSK-3β signaling in the regulation of OS is self-evident.

A number of reports have documented that the expression of *PHLDA3* is low in many different types of tumors. For example, *PHLDA3* is inactivated by both LOH and methylation in various neuroendocrine tumors (NETs), such as Lung NETs and PanNETs [33]. Typically, miRNAs can directly dock to the 3′ UTR of the targeted mRNA, inhibiting translation or causing mRNA degradation [34,35]. Hence, we adopted bioinformatics tools to identify a docking site for miR-19a-3p in the *PHLDA3* 3’-UTR. Interestingly, the data confirmed that miR-19a-3p represses *PHLDA3* expression. As a member of the known oncomiR-17-92 cluster [36], miR-19a-3p is frequently dysregulated and has pro-cancerous and pro-proliferative roles in many tumors, including gastric cancer [37], hepatocellular carcinoma [38], and breast cancer [39]. Overexpression of miR-19a-3p was identified in OS cells, and silencing of miR-19a-3p promoted its chemosensitivity via escalating the expression of tumor repressor PTEN [21]. Herein, we found that overexpressing miR-19a-3p increased OS cell proliferation, metastasis, and drug resistance. Thus, miR-19a-3p might have pro-tumorigenic effects in OS by inhibiting *PHLDA3* expression.

## 5. Conclusion

Overall, our findings show that *PHLDA3*, a true target of miR-19a-3p, is less expressed in OS tissue specimens and suppresses the proliferation, malignant and chemoresistance biological behavior by affecting the Akt/GSK-3β signaling cascade. Furthermore, *PHLDA3* may be considered an independent predictor of poor prognosis, as well as a prospective therapeutic target for OS patients.

## Data Availability

Data will be made available on request to the corresponding authors (http://dx.doi.org/10.1080/21655979.2022.2031404).

## Research highlights

1. *PHLDA3* expression was low in osteosarcoma patients, and low content was linked to poor prognosis.
2. *PHLDA3* suppressed osteosarcoma cell proliferation, migration, and chemoresistance via regulating the Akt/GSK-3β signaling.
3. miR-19a-3p inhibits *PHLDA3* expression in osteosarcoma.

## References

[1] Wu Y, Xie Z, Chen J, et al. Circular RNA circTADA2A promotes osteosarcoma progression and metastasis by sponging miR-203a-3p and regulating CREB3 expression. Mol Cancer. 2019;18(1):73. 30940151 10.1186/s12943-019-1007-1 PMC6444890

[2] Ma J, Gao W, Gao J. sCLU as prognostic biomarker and therapeutic target in osteosarcoma. Bioengineered. 2019;10(1):229–239. 31117872 10.1080/21655979.2019.1621136 PMC6592366

[3] Shen S, Yao, T, Xu, Y, et al. CircECE1 activates energy metabolism in osteosarcoma by stabilizing c-Myc. Mol Cancer. 2020;19(1):151. 33106166 10.1186/s12943-020-01269-4 PMC7586679

[4] Zheng D, Liu W, Xie W, et al. AHA1 upregulates IDH1 and metabolic activity to promote growth and metastasis and predicts prognosis in osteosarcoma. Signal Transduct Target Ther. 2021;6(1):25. 33468990 10.1038/s41392-020-00387-1 PMC7815748

[5] Wang X, Qin G, Liang X, et al. Targeting the CK1alpha/CBX4 axis for metastasis in osteosarcoma. Nat Commun. 2020;11(1):1141. 32111827 10.1038/s41467-020-14870-4 PMC7048933

[6] Tonella L, Pala V, Ponti R, et al. Prognostic and predictive biomarkers in stage III melanoma: current insights and clinical implications. Int J Mol Sci. 2021;22(9):4561. 33925387 10.3390/ijms22094561 PMC8123895

[7] Fang J, Ge X, Xu W, et al. Bioinformatics analysis of the prognosis and biological significance of HMGB1, HMGB2, and HMGB3 in gastric cancer. J Cell Physiol. 2020;235(4):3438–3446. 31621076 10.1002/jcp.29233 PMC13482706

[8] Leung SW, Chia-Jung C, Tsui-Chin H, et al. An integrated bioinformatics analysis repurposes an antihelminthic drug niclosamide for treating HMGA2-overexpressing human colorectal cancer. Cancers (Basel). 2019;11(10):1482. 10.3390/cancers11101482 PMC6826424 31581665

[9] Huang S, Zhao J, Song J, et al. Interferon alpha-inducible protein 27 (IFI27) is a prognostic marker for pancreatic cancer based on comprehensive bioinformatics analysis. Bioengineered. 2021;12(1):8515–8528. 34592906 10.1080/21655979.2021.1985858 PMC8806992

[10] Fan L, Cao X, Lei Y. MicroRNA miR-23b-3p promotes osteosarcoma by targeting ventricular zone expressed PH domain-containing 1 (VEPH1)/phosphatidylinositol 3-kinase/protein kinase B (PI3K/AKT) pathway. Bioengineered. 2021;12(2):12568–12582. 34903122 10.1080/21655979.2021.2010383 PMC8810025

[11] Keremu A, Maimaiti X, Aimaiti A, et al. NRSN2 promotes osteosarcoma cell proliferation and growth through PI3K/Akt/MTOR and Wnt/beta-catenin signaling. Am J Cancer Res. 2017;7(3):565–573. 28401012 PMC5385644

[12] Kawase T, Ohki R, Shibata T, et al. PH domain-only protein PHLDA3 is a p53-regulated repressor of Akt. Cell. 2009;136(3):535–550. 19203586 10.1016/j.cell.2008.12.002

[13] Ohki R, Saito K, Chen Y, et al. PHLDA3 is a novel tumor suppressor of pancreatic neuroendocrine tumors. Proc Natl Acad Sci U S A. 2014;111(23):E2404–13. 24912192 10.1073/pnas.1319962111 PMC4060701

[14] Ma S, Quan P, Yu C, et al. PHLDA3 exerts an antitumor function in prostate cancer by down-regulating Wnt/beta-catenin pathway via inhibition of Akt. Biochem Biophys Res Commun. 2021;571:66–73. 34303965 10.1016/j.bbrc.2021.07.038

[15] Takikawa M, Ohki R. A vicious partnership between AKT and PHLDA3 to facilitate neuroendocrine tumors. Cancer Sci. 2017;108(6):1101–1108. 28295876 10.1111/cas.13235 PMC5480075

[16] F RM, AV Valoyes M, G Do Nascimento R, et al. Prognostic and predictive value of Pleckstrin homology-like domain, family A family members in breast cancer. Biomark Med. 2020;14(16):1537–1552. 33179538 10.2217/bmm-2020-0417

[17] Zhang D, Lin J, Chao Y, et al. Regulation of the adaptation to ER stress by KLF4 facilitates melanoma cell metastasis via upregulating NUCB2 expression. J Exp Clin Cancer Res. 2018;37(1):176. 30055641 10.1186/s13046-018-0842-z PMC6064624

[18] Zhou K, Chen J, Wu J, et al. Profilin 2 promotes proliferation and metastasis of head and neck cancer cells by regulating PI3K/AKT/beta-catenin signaling pathway. Oncol Res. 2019;27(9):1079–1088. 31122311 10.3727/096504019X15579146061957 PMC7848265

[19] Cheng J, Huang Y, Zhang X, et al. TRIM21 and PHLDA3 negatively regulate the crosstalk between the PI3K/AKT pathway and PPP metabolism. Nat Commun. 2020;11(1):1880. 32312982 10.1038/s41467-020-15819-3 PMC7170963

[20] Luo T, Zhou X, Jiang E, et al. Osteosarcoma cell-derived small extracellular vesicles enhance osteoclastogenesis and bone resorption through transferring MicroRNA-19a-3p. Front Oncol. 2021;11:618662. 33842319 10.3389/fonc.2021.618662 PMC8029976

[21] Zhang B, Liu Y, Zhang J. Silencing of miR-19a-3p enhances osteosarcoma cells chemosensitivity by elevating the expression of tumor suppressor PTEN. Oncol Lett. 2019;17(1):414–421. 30655782 10.3892/ol.2018.9592 PMC6313165

[22] Saito M, Sada A, Fukuyo M, et al. PHLDA3 is an important downstream mediator of p53 in squamous cell carcinogenesis. J Invest Dermatol. 2021. DOI:10.1016/j.jid.2021.09.007. 34592332

[23] Lee CG, Kang YJ, Kim HS, et al. Phlda3, a urine-detectable protein, causes p53 accumulation in renal tubular cells injured by cisplatin. Cell Biol Toxicol. 2015;31(2):121–130. 25809501 10.1007/s10565-015-9299-4

[24] Lee CG, Kim JG, Kim HJ, et al. Discovery of an integrative network of microRNAs and transcriptomics changes for acute kidney injury. Kidney Int. 2014;86(5):943–953. 24759152 10.1038/ki.2014.117

[25] Qiao M, Wu M, Shi R, et al. PHLDA3 impedes somatic cell reprogramming by activating Akt-GSK3beta pathway. Sci Rep. 2017;7(1):2832. 28588267 10.1038/s41598-017-02982-9 PMC5460190

[26] Liu J, Liu X, Hui X, et al. Novel role for pleckstrin homology-like domain family a, member 3 in the regulation of pathological cardiac hypertrophy. J Am Heart Assoc. 2019;8(16):e011830. 31426686 10.1161/JAHA.118.011830 PMC6759890

[27] Liu L, Dai Y, Chen J, et al. Maelstrom promotes hepatocellular carcinoma metastasis by inducing epithelial-mesenchymal transition by way of Akt/GSK-3beta/Snail signaling. Hepatology. 2014;59(2):531–543. 23929794 10.1002/hep.26677

[28] An P, Chen F, Li Z, et al. HDAC8 promotes the dissemination of breast cancer cells via AKT/GSK-3beta/Snail signals. Oncogene. 2020;39(26):4956–4969. 32499521 10.1038/s41388-020-1337-x

[29] Sun J, Tang Q, Gao Y, et al. VHL mutation-mediated SALL4 overexpression promotes tumorigenesis and vascularization of clear cell renal cell carcinoma via Akt/GSK-3beta signaling. J Exp Clin Cancer Res. 2020;39(1):104. 32513235 10.1186/s13046-020-01609-8 PMC7278163

[30] Zhang S, Wang J, Chen T, et al. alpha-Actinin1 promotes tumorigenesis and epithelial-mesenchymal transition of gastric cancer via the AKT/GSK3beta/beta-Catenin pathway. Bioengineered. 2021;12(1):5688–5704. 34546849 10.1080/21655979.2021.1967713 PMC8806412

[31] Zhang Y, Cheng H, Li W, et al. Highly-expressed P2X7 receptor promotes growth and metastasis of human HOS/MNNG osteosarcoma cells via PI3K/Akt/GSK3β/β-catenin and mTOR/HIF1α/VEGF signaling. Int J Cancer. 2019;145(4):1068–1082. 30761524 10.1002/ijc.32207 PMC6618011

[32] Liang C, Yu, X, Xiong, N, et al. Pictilisib enhances the antitumor effect of doxorubicin and prevents tumor-mediated bone destruction by blockade of PI3K/AKT pathway. Front Oncol. 2020;10:615146. 33659212 10.3389/fonc.2020.615146 PMC7917262

[33] Chen Y, and Ohki R. p53-PHLDA3-Akt network: the key regulators of neuroendocrine tumorigenesis. Int J Mol Sci. 2020;21(11):4098. 10.3390/ijms21114098 PMC7312810 32521808

[34] Lin J, Liao, S, Li, E, et al. circCYFIP2 acts as a sponge of mir-1205 and affects the expression of its target gene E2F1 to regulate gastric cancer metastasis. Mol Ther Nucleic Acids. 2020;21:121–132. 32526476 10.1016/j.omtn.2020.05.007 PMC7286931

[35] Ren R, Yuan Z, Xu Z. miRNA-144 targeting DNAJC3-AS1 reverses the resistance of the breast cancer cell line Michigan Cancer Foundation-7 to doxorubicin. Bioengineered. 2021;12(2):9885–9892. 34895046 10.1080/21655979.2021.1999373 PMC8810046

[36] Sun HX, Yang Z-F, Tang W-G, et al. MicroRNA-19a-3p regulates cell growth through modulation of the PIK3IP1-AKT pathway in hepatocellular carcinoma. J Cancer. 2020;11(9):2476–2484. 32201518 10.7150/jca.37748 PMC7066004

[37] Qiao F, Gong P, Song Y, et al. Downregulated PITX1 modulated by MiR-19a-3p promotes cell malignancy and predicts a poor prognosis of gastric cancer by affecting transcriptionally activated PDCD5. Cell Physiol Biochem. 2018;46(6):2215–2231. 29734189 10.1159/000489590

[38] Jiang XM, Yu X-N, Liu -T-T, et al. microRNA-19a-3p promotes tumor metastasis and chemoresistance through the PTEN/Akt pathway in hepatocellular carcinoma. Biomed Pharmacother. 2018;105:1147–1154. 30021351 10.1016/j.biopha.2018.06.097

[39] Lee S, Lee, H, Bae, H, et al. Epigenetic silencing of miR-19a-3p by cold atmospheric plasma contributes to proliferation inhibition of the MCF-7 breast cancer cell. Sci Rep. 2016;6(1):30005. 27445062 10.1038/srep30005 PMC4956745

